# The Contribution of Home, Neighbourhood and School Environmental Factors in Explaining Physical Activity among Adolescents

**DOI:** 10.1155/2009/320372

**Published:** 2009-09-14

**Authors:** Leen Haerens, Mietje Craeynest, Benedicte Deforche, Lea Maes, Greet Cardon, Ilse De Bourdeaudhuij

**Affiliations:** ^1^Research Foundation-Flanders, Department of Movement and Sports Sciences, Ghent University, Belgium; ^2^Department of Movement and Sports Sciences, Ghent University, Belgium; ^3^Department of Public Health, Ghent University, Belgium

## Abstract

The present study aimed at investigating the influence of home, neighbourhood and school environmental factors on adolescents' engagement in self-reported extracurricular physical activity and leisure time sports and on MVPA objectively measured by accelerometers. Environmental factors were assessed using questionnaires. Gender specific hierarchical regression analyses were conducted, with demographic variables entered in the first block, and environmental, psychosocial factors and interactions terms entered in the second block. Participation in extracurricular activities at school was positively related to the number of organized activities and the provision of supervision. Perceived accessibility of neighborhood facilities was not related to engagement in leisure time sports, whereas the availability of sedentary and physical activity equipment was. Findings were generally supportive of ecological theories stating that behaviors are influenced by personal and environmental factors that are constantly interacting.

## 1. Introduction

Many adolescents are insufficiently active for enhanced health and weight control [1]. In order to effectively promote physical activity among adolescents, it is necessary to understand factors that influence adolescents' physical activity levels [2].

 Ecological models such as the social ecological theory [3] and the social cognitive theory [4] recognize the importance of simultaneously investigating demographic, psychosocial, and environmental factors to understand physical activity behaviors [5]. In addition, it has been demonstrated that relationships between personal or environmental factors and physical activity levels differ according to the type of physical activity behavior and the context in which it takes place [6].

 Although a recent review showed that participation in extracurricular activities at school considerably contributes to adolescents overall engagement in physical activities of moderate-to-vigorous intensity [7], few studies looked at this specific type of behavior typically related to the school context [2, 8]. At the same time, schools are considered as preferred environments for promoting physical activity among adolescents [9, 10]. Schools offer many opportunities for adolescents to engage in physical activities (i.e., physical education classes, extracurricular physical activities, and recess periods) and adolescents spend large amounts of time at school. Although it is recommended to promote physical activity during physical education classes, physical education time is limited and even the best school physical education programs do not provide enough physical activity to meet health-related recommendations [11]. Therefore, it has been suggested that the organization of physical activities at school outside physical education classes, such as during the lunch breaks, during recesses, and during after school hours, might be important to reach the recommended daily physical activity time of one hour. One study [9] in American middle schools found that schools that organize extracurricular activities might increase physical activity levels among boys (grades 6–8). The same study pointed out that the objectively measured school environment (area type, area size, supervision, equipment, improvements) explains the largest amount of variance in the number of boys and girls engaging in MVPA at school. Boys were most active on outdoor courts with high levels of supervision or when both equipment and supervision were provided. Both boys and girls were more active when schools had improvements (e.g., basketball hoops, volleyball nets, tennis courts) and high levels of supervision. Two studies among Norwegian adolescents showed that self-reported activity levels during recess were positively related to the number of available outdoor spaces [12, 13] and the availability of playground equipment [13]. However, the authors argued that “more in-depth research simultaneously addressing the impact of individual, sociocultural, environmental factors, and the interaction between them is needed to get a better understanding of activity levels at school” [12].

 Therefore, the first purpose of this explorative study was to investigate the relationship between environmental features of the school environment such as accommodation and supervision and participation in extracurricular physical activities at school, while simultaneously including psychosocial determinants.

 Next to school-based activities, also leisure time sports take a significant place in the activity culture of many adolescents [14]. For engagement in leisure time sports, the home and neighborhood environment might be important contexts to consider.

 Studies in adults extensively investigated the relationship between the neighborhood environment (e.g., access to facilities) and participation in leisure time physical activity [15–17]. The results of a recent review [8] showed that among adolescents, only four neighborhood environmental factors (access to facilities, availability of facilities, availability of equipment, crime incidence) were examined in more than three samples. Availability and accessibility of equipment and facilities were identified as unrelated [8]. The same review furthermore revealed that only one adolescent study looked at the influence of distance to destinations and residential density, suggesting that studies including these variables are needed.

 With regard to the home environment, most prior studies explored sociocultural (i.e., family structure, modelling and support from family and friends) correlates of physical activity among adolescents [8]. However, physical environmental factors were less frequently investigated. Only seven studies investigated the effects of availability of physical activity equipment and most of these studies were conducted in the US and Canada [8]. Availability of exercise equipment was found to be unrelated to adolescents' physical activity levels. Additionally, none of these studies included a measure of availability of sedentary equipment (e.g., play stations and televisions).

 Therefore, a second purpose of this explorative study was to investigate the relationship between adolescents' engagement in leisure time sports and neighborhood and home environmental features, while simultaneously including psychosocial determinants and the interactions between the two. Finally, as health-related guidelines specifically address engagement in physical activity of moderate-to-vigorous intensity (MVPA), the final purpose of this study was to investigate psychosocial and environmental correlates of objectively measured MVPA by means of accelerometers.

## 2. Methods

### 2.1. Participants

Participants were recruited from four randomly selected middle schools offering technical-vocational education in West-Flanders (Belgium). Parents of all students in seventh and eight grade (*n* = 667) received a letter seeking informed consent for each child to complete measurements. Parents of 634 (95%) adolescents gave permission for their child to participate in this study. Of those adolescents, 523 completed all questionnaires; missing data were due to absence on the day of measurements or questionnaires filled out inaccurately, yielding in a final response rate of 78%. From each of the four schools, one class of seventh graders was randomly selected for more in-depth physical activity measurements with accelerometers, which resulted in a subsample of 62 adolescents. The study protocol was approved by the Ethical Committee of the Ghent University.

### 2.2. Measures

#### 2.2.1. Questionnaire

Data on demographics like gender, birth date, and occupation of father and mother were collected in the first part of the questionnaire. An estimate of higher and lower social economic status (SES) was obtained by classifying occupation of father and mother into white and blue collar [18].

 Participation in extracurricular activities at school and leisure time sports was determined in a second part of the questionnaire using a selection of questions from the Flemish Physical Activity Questionnaire (FPAQ, see Supplementary Material available online at doi:10.1155/2009/320372). One question addressed time spent in extracurricular physical activities at school (question 4, see Supplementary Material). Three questions asked for frequency and duration of time spent in leisure time sports (question 7, see Supplementary Material).

 Validity and reliability of a computerized version of the questionnaire used in the present study was investigated in a separate study with different participants aged 12–18 [19]. Moderate-to-high reliability of the indexes derived from the FPAQ was reported. For all types of physical activity test-retest, ICC's exceeded 0.70. To obtain validity measures, data from questionnaires were correlated to data derived from accelerometers. Pearson correlations were significant and ranged between 0.43 (total activity levels) and 0.78 (minutes spent in vigorous activities), indicating acceptable validity of the instrument used in the present study [19].

 Ecological models recognize the importance of the interactions between personal-level factors and the environment [5]. To investigate these interactions, psychosocial determinants were assessed in a third part of the questionnaire. Measures of students' general-affective attitudes, that is, social support, self-efficacy, perceived benefits, and barriers, were assessed by 29 items with a 5-point scale. Questions were selected and adopted from previous studies with adolescents and adults [20–22]. General-affective attitudes (4 items) toward physical activity were assessed using bipolar adjectives. Participants were asked whether sports and physical activity are “not pleasant-pleasant,” “bad-good,” “healthy-unhealthy,”, and “dangerous-safe” (Cronbach's *α* = 0.79). Social support (4 items) was assessed by asking respondents how frequently their parents, brothers and sisters, friends, and teachers encouraged them to be physically active (Cronbach's *α* = 0.79). Self-efficacy (2 items) was measured by asking how easy or difficult it is to be active at their school or at their home (Cronbach's *α* = 0.38). Perceived benefits and barriers with regard to physical activity were investigated by asking respondents to rate their agreement with possible effects of sports and physical activity (8 items: weight and physical appearance, health and fitness, social interaction, pleasure, competition, stress and depression, admiration of others, relaxation from (school) work, Cronbach's *α* = 0.85), and the frequency with which barriers prevented them from exercising (11 items: lack of time, lack of discipline, lack of interest, health problems, personal problems, not skilled enough, too expensive, no transportation, not liking to sweat, fear of being laughed at, lack of facilities at school, Cronbach's *α* = 0.88). For measures of psychosocial determinants test-retest ICC's significantly ranged between 0.61 and 0.90.

#### 2.2.2. Accelerometers

Physical activity levels were also assessed using accelerometers (model 7164, Computer Science Application, Inc., Shalimar, Fla, USA). Accelerometers are considered as valid and reliable tools for assessment of physical activity among adolescents [23]. Accelerometer measurements could only be conducted in a subsample of one class per school (*n* = 62 adolescents) because given the expense of these instruments, only a limited number of instruments were available. Independent sample *t*-tests with self-reported physical activity levels as dependent variables indicated that there were no significant differences between those students with and without accelerometer data (all *t* ≤ 0.9).

 Adolescents wore the accelerometer during six days above the right hipbone, underneath the clothes. Accelerometers measured activity counts in epoch times of one minute. Accelerometer data were used to determine engagement in physical activity of moderate to vigorous intensity (MVPA). In agreement with most recently published guidelines, the cut-off point for MVPA was ≥3200 counts per minute [23, 24].

#### 2.2.3. Questionnaire for the Home Environment-Related to Physical Activity

To measure potential environmental correlates of physical activity among adolescents, a modified version of a questionnaire validated in adults was used [15]. The scale composition, scale items, response categories, reliability, and validity data are all represented in Table 1. The first part of the questionnaire asked for convenience of neighborhood facilities for adolescents. Five items were rated on a five-point scale from “1–5 minutes” cycling from home to “more than 30 minutes” cycling from home. To measure neighborhood residential density adolescents rated on a three-point scale (none-some-much), how many detached single-family residences, row houses, and apartments there were in their neighborhood. The number of TV's, computers, and playstations was questioned to get a measure of the availability of sedentary equipment at home. Finally, home availability of physical activity equipment (13 items) was questioned (see Table 1). Test-retest reliability was analyzed by subjects completing the questionnaires twice within a 2-week interval. To test validity, all parents of participating pupils were contacted by telephone to verbally answer the same questionnaire as their child. The lowest ICC was found for residential density (0.49), for all other indexes, ICC's significantly ranged between 0.63 and 0.95. Validity coefficients significantly ranged between 0.46 and 0.95.

#### 2.2.4. The Physical Activity-Related School Environment

A questionnaire for measuring the school environment related to physical activity was completed by one teacher at each school (*n* = 4). Accessibility of sports facilities and sports materials availability of supervision and extracurricular activities (during breaks and after school hours) was measured using a three-point answering scale (daily-weekly-never).

### 2.3. Statistical Analyses

All analyses were conducted with SPSS 12.0. Preliminary analyses consisted of descriptive statistics of sample characteristics.

 Multiple linear hierarchical regression analyses with extracurricular physical activity, leisure time sports, and MVPA as dependent variables were conducted to investigate the relationship between personal, environmental factors, and these physical activity measurements. All analyses were controlled for age and SES by entering these factors into the first block. In the first series of regression analyses (model 1), neighborhood (convenience of facilities), home (sedentary and sports equipment), and school (availability of physical activities, accessibility of sports accommodation and sports materials, supervision) environmental variables were entered into the second block. Due to its low reliability (0.49) residential density was excluded from the analyses. For analyses on time spent in extracurricular physical activity, only school-related environmental variables were included. For leisure time sports, only neighborhood and home environmental factors were entered into the second block. For MVPA school, neighborhood and home environmental factors were included. In the second series of regression analyses (model 2) psychosocial determinants (attitude, self-efficacy, social support, perceived benefits, and perceived barriers) were entered into the second block. In the third series of regression analyses (model 3), interactions between environmental and psychosocial determinants were entered into the second block.

 To investigate the interaction effects, the product of two variables was computed after these were mean centred. In a first phase, hierarchical regression analyses with one environmental correlate, and the interaction terms between that specific correlate and the psychosocial determinants were conducted, to test which variables needed to be included in the final models. In a second phase, only significant correlates were included in the regression analyses (see Tables 3–7). Finally, all variables were added together to estimate the total variance explained.

 In all of the models, variation inflation factors were below 10, indicating that there were no problems of multicollinearity [25]. Previous studies showed gender differences in levels of physical activity [1, 26] or intervention effects [27–29]. Preliminary analyses furthermore revealed differences in physical activity levels and correlates of physical activity for boys and girls of the present sample. In addition, gender-specific analyses are recommended [26]. Therefore, all analyses were conducted in boys and girls separately. However, due to the small sample size (*n* = 62), analyses on MVPA were conducted in boys and girls together. The value *P* ≤ .05 was considered as statistically significant.

## 3. Results

The descriptive characteristics of the sample are presented in Table 2.

### 3.1. Self-Reported Extracurricular Physical Activity at School

Hierarchical regression analyses on participation in extracurricular physical activities are presented in Table 3 for boys and Table 4 for girls.

 Among boys, the total model explained 28% of the variance in participation in extracurricular physical activity. In the first model, school environmental factors accounted for 6% of the variance. Availability of extra physical activities was the only significant correlate within the second block. In the second model, the psychosocial determinants explained 19% of the variance. Self-efficacy was the only significant correlate within the second block. In the third model, the interaction terms explained 10% of the variance. The interaction term between supervision and perceived benefits was significant.

 Among girls, the total model explained 17% of the variance in participation in extracurricular physical activity. In the first model, school environmental factors accounted for 7% of the variance. Supervision was the only significant correlate within the second block. In the second model, psychosocial determinants explained 7% of the variance. Self-efficacy and perceived barriers were significant correlates of extracurricular physical activity. In the third model, the interactions terms explained 4.0% of the variance. The interaction term between organized physical activities and perceived benefits was significant.

### 3.2. Self-Reported Leisure Time Sports

Hierarchical regression analyses on self-reported leisure time sports levels are presented in Table 5 for boys and Table 6 for girls.

 Among boys, the entire model explained 32% of the variance in leisure time sports. None of the environmental variables correlated significantly with leisure time sports. The second model with psychosocial determinants explained 19% of the variance. Perceived benefits were significantly positively related to leisure time sports among boys. In the third block, interaction terms significantly explained 23% of the variance. The interactions terms between sedentary equipment and social support and sedentary equipment and barriers were significant.

 Among girls, the entire model explained 27% of the variance in leisure time sports. In the first model, only 1% was explained by the second environmental block. Availability of PA equipment was the only significant environmental correlate. In the second model, psychosocial determinants explained 21% of the variance. Attitude and self-efficacy were significantly positively related to leisure time sports among girls. In the third block, the interaction terms significantly explained 7% of the variance. The interaction term between convenience of facilities and barriers was significant.

### 3.3. MVPA Measured with Accelerometers

Hierarchical regression analyses on MVPA are presented in Table 7. The entire model explained 51% of the variance in MVPA. In the first model, environmental factors explained 1% of the variance in MVPA. In the second model, the psychosocial determinants explained 18% of the variance. Perceived benefits were positively related to MVPA, whereas the attitude was negatively correlated to MVPA. In the third model, interactions terms explained 10.6% of the variance. The interaction term between supervision and self-efficacy was significant.

## 4. Discussion

The first purpose of this explorative study was to investigate the relationship between environmental features of the school environment and participation in extracurricular activities at school. The time spent in extracurricular activities at school contributes considerably to overall physical activity levels [7]. Hence, in order to be able to design effective school-based intervention to increase this type of behavior, it is important to investigate influencing factors. Findings of the present study showed that adolescents' engagement in extracurricular physical activity at school was positively related to the availability of organized activities at school. For girls, significant interactions with perceived benefits were found, with stronger correlations between organized activities and engagement in extracurricular activities among girls reporting more benefits. In contrast to our results, the organization of school sports was defined as unrelated to adolescents' physical activity levels in a recent review [8]. On the other hand and in line with our findings, environmental intervention studies already showed that offering extra physical activities at school appeared to be an effective strategy for increasing physical activity engagement at school (e.g., [29]). Results from the present study furthermore revealed that the provision of supervision was also positively related to participation in extracurricular activities. Again, significant interactions with perceived benefits were found, with stronger correlations between supervision and engagement in extracurricular activities among boys reporting more benefits. The importance of supervision was also exposed in a study among American adolescents showing that boys were more active when supervision was provided [9]. In contrast to the study among Norwegian adolescents [12, 13], the present study found that access to accommodation and sports materials were both unrelated to participation in extracurricular activities at school.

 A second purpose of the present study was to investigate correlates of participation in leisure time sports in a sample of Flemish adolescents. For adolescents, engagement in sport activities can be considered as an important leisure time activity [14], making it highly relevant to look at environmental correlates for this specific behavior in this age group. For engagement in leisure time sports, the neighborhood environment is particularly important to consider. In the present study only, one neighborhood environmental factor, namely, the perceived convenience of facilities for adolescents was included. In the American or Canadian context, availability of facilities for adolescents [8] was found to be unrelated adolescents' engagement in physical activities. On the other hand, studies in adults found positive associations between perceived convenience of facilities and leisure time physical activity [17]. The results of the present study, however, revealed that perceived convenience of facilities was unrelated to leisure time sports among adolescents. However, among girls an interaction with barriers for physical activity was found, with stronger correlations between convenience of facilities and leisure time sports among girls perceiving more barriers.

 Clearly, future research should include more extensive measurements of those neighborhood factors (i.e., access to facilities, influence of parks, etc.) that might be more closely related to engagement in leisure time sports. A recently published study of Canadian adolescents showed that beside the number of available recreation facilities, access to facilities might also be important to consider [30]. Additionally, some studies have recently shown that the objectively measured availability and accessibility of physical activity facilities [31–33] might be more important when compared to perceived environmental factors. Future studies should, therefore, include objective measurements of the actual neighborhood environment such as the application of geographic information systems (GISs) data [34] in a European adolescent population.

 Participation in leisure time sports might also be influenced by the home environment. The availability of equipment (e.g., Playstation) that facilitates sedentary behavior might discourage adolescents from going outside and engaging in sports activities. Results showed that the availability of sedentary equipment was indeed negatively related to participation in leisure time sports, but only among boys. In addition, interactions with psychosocial determinants such as barriers should be considered. Negative correlations between sedentary equipment and leisure time sports were only found among boys perceiving fewer barriers to be physically active. Studies have shown that boys more frequently engage in screen time compared to girls [35], and playing computer games is considered as a rather masculine activity [36]. Therefore, it is plausible that boys more often use sedentary equipment when it is available; whereas the availability of sedentary equipment might be less attractive to girls. Future research should elaborate on these findings by adding questions on time spent using sedentary equipment or rules regarding the use of sedentary equipment at home.

 Studies among adults revealed positive relationships between the availability of physical activity equipment in the home environment and activity levels among men and women [37]. In the present sample of adolescents, the availability of physical activity equipment was also positively related to engagement in leisure time sports among girls. Among boys, interactions with self-efficacy were found, with stronger correlations between equipment and leisure time sports among boys reporting higher levels of self-efficacy. However, in adult studies [37], the argument is raised that people who engage in sport activities more regularly might be more likely to purchase physical activity equipment. Therefore, also among adolescents, engagement in leisure time sports might more likely be an antecedent rather than a consequence of availability of sports equipment in the home environment. In addition, for adolescents, the availability of sports equipment might also be closely related to the activity levels of other family members such as parents who are known to influence adolescents' activity levels [8].

 Participation in MVPA is essential for improved health. In line with findings from Jago et al. [38], most of the environmental factors were not associated with participation in MVPA. Only the interaction between self-efficacy and supervision was significant, with stronger correlations between supervision and MVPA among those reporting higher levels of self-efficacy. However, research that further investigates the relationships between environmental factors and objectively measured MVPA in larger samples is needed to be conclusive.

 According to ecological models of behavior change, the environment does not influence behavior separate from individual determinants [3, 4]. Previous intervention studies have shown favorable changes in physical activity levels as a result of the combination of environmental strategies with personal interventions [27, 28, 39]. The results of the present study revealing several significant interactions between personal and environmental variables are supportive of such multicomponent intervention designs.

 Some limitations of the present study need to be addressed. First, the questionnaire to measure school level variables was not validated yet; therefore, conclusions concerning school environmental factors should be treated with considerable caution. The development of a comprehensive valid and reliable questionnaire to measure school environmental factors by use of self-reports is a priority for future research. Although it is the strength of the present study that physical activity was also measured more objectively with accelerometers, the subsample in which these measurements occurred was very small. Hence, most of the conclusions in the present study were based on self-reported physical activity measures and although validated questionnaires were used, this is a limitation. Although the questionnaire on psychosocial determinants was already extensive, no questions on social norms were included. Especially among adolescents, apart from social support, social norms (i.e., influence of peers) might be important to consider [40]. Furthermore, given the cross-sectional design of the present study, no causal conclusions can be drawn. Although cross-sectional studies are necessary to understand the relationships between environmental factors and physical activity levels, prospective studies that further explore the causal relationships between the change in personal, intrapersonal, and environmental factors and the change in physical activity levels are needed to formulate recommendations for physical activity promotion.

## 5. Conclusion

This explorative study provided initial insight into the relationship between school environmental factors and participation in extracurricular physical activity in a sample of European adolescents. The findings showed that increased availability of extracurricular activities and supervision are related to greater participation in extracurricular activities among adolescent boys and girls. School-based intervention studies that investigate the effects of such strategies are recommended. The findings showed that perceived convenience of neighborhood facilities was unrelated, whereas the availability of sedentary and physical activity equipment at home was related to time spent in leisure time sports. Overall, findings were supportive of ecological theories stating that behaviors are influenced by personal and environmental factors that are constantly interacting [4].

## Supplementary Material

The appendix provides an overview of the selection of questions from the Flemish Physical Activity Questionnaire (Philippaerts et al., 2006) that were used for the present study.

## Figures and Tables

**Table 1:** Summary of environmental scales, items, response categories, intraclass correlations (reliability), and pearsons' correlations (Validity).

Scale (composition)	Item	Response category	ICC	Pearson r
*Convenience of facilities for adolescents* (6 items)	About how long would it take to get from your home to: school, the sports room, the football court, the cinema, the playground and the swimming pool?	5-point scale ^(a)^	0.63^‡^	0.46*

*Density* (3 items)	How common is each type of residence in your neighborhood: Detached single-family residence, Row house, Apartment	3-point scale^(b)^	0.49^†^	0.55^†^

*Sedentary equipment* (3 items)	How many computers/TV's do you have at home? Do you have a playstation?	5-point scale^(c)^ Yes/No	0.95^‡^	0.95^‡^

*Physical activity equipment* (13 items)	Indicate which items you have at home: Bicycle, running shoes, trampoline, table tennis, swimming pool, fitness equipment, step, roller blades, tennis/badminton rackets, basketball goal, rope, football, skateboard	Yes/No	0.88^‡^	0.91^‡^

^(a)^Five-point scale: 1–5, 6–10, 11–20, 21–30, >30 minutes; ^(b)^Threethree-point scale: none, much, a lot; ^(c)^Fivefive-point scale: 0,1, 2,3, 4, >4. *P ≤ .05, ^†^ *P* ≤ .01, ^‡^ *P* ≤ .001.

**Table 2:** Descriptive characteristics (% or means and standard deviations) for the total sample of boys and girls.

	Total sample	Boys	Girls
*(n)*	*(523)*	*(197)*	*(326)*
Age (years)	12.7 ± 0.6	12.8 ± 0.6	12.6 ± 0.6
% high SES	49.8 ± 50.0	55.9 ± 49.9	46.9 ± 50.0
Attitude^(a)^	4.2 ± 0.6	4.2 ± 0.6	4.1 ± 0.6
Self-efficacy^(a)^	3.7 ± 0.8	3.8 ± 0.7	3.6 ± 0.7
Social support^(a)^	2.2 ± 0.9	2.3 ± 0.9	2.2 ± 0.9
Perceived benefits^(a)^	3.5 ± 0.7	3.6 ± 0.7	3.5 ± 0.7
Perceived barriers^(a)^	2.0 ± 0.6	1.9 ± 0.7	2.0 ± 0.6
*Self-reported physical activity*			
Leisure time sports (minutes/day)	31.2 ± 34.2	39.0 ± 37.9	26.5 ± 31.0
Extracurricular Physical Activity (minutes/day)	4.4 ± 8.8	7.6 ± 10.8	2.5 ± 6.6
*Accelerometers*			
*(n)*	*(62)*	*(12)*	*(50)*
MVPA (minutes/day)	17.1 ± 13.1	29.2 ± 14.8	14.2 ± 11.0

^(a)^On a scale 1–5, from negative to positive.

**Table 3:** Hierarchical regression of the physical school environment on extracurricular physical activities at school among boys.

	*R* ^2^	*R*_change_^2^	*F*_change_	
*Block 1*	0.02	0.02	1.79	*Beta 1*
SES				0.08
Age				−0.12
*Block 2-Model 1*	0.08	0.06	3.93^†^	*Beta 2*
Extra physical activities				0.24^‡^
Supervision				0.01
Access to accommodation at school				0.02
*Block 2-Model 2*	0.21	0.19	9.22^‡^	*Beta 2*
Attitude				−0.01
Self-efficacy				0.35^‡^
Social support				0.05
Perceived benefits				0.13
Perceived barriers				−0.08
*Block 2-Model 3*	0.12	0.10	7.55^‡^	*Beta 2*
Supervision				0.16*
Perceived benefits				0.24^‡^
Supervision*benefits				0.17*

*P ≤ .05, ^†^ *P* ≤ .01, ^‡^ *P* ≤ .001.

**Table 4:** Hierarchical regression of the school environment on extracurricular physical activities at school among girls.

	*R* ^2^	*R*_change_^2^	*F*_change_	
*Block 1*	0.02	0.02	3.14*	*Beta 1 *
SES				0.07
Age				−0.13*
*Block 2-Model 1*	0.09	0.07	12.34^‡^	*Beta 2*
Extra physical activities				0.06
Supervision				0.25^‡^
*Block 2-Model 2*	0.09	0.07	4.79^‡^	*Beta 2*
Attitude				−0.09
Self-efficacy				0.16*
Social support				0.08
Perceived benefits				0.12
Perceived barriers				−0.14*
*Block 2-Model 3*	0.06	0.04	5.04^†^	*Beta 2*
Extra physical activities				0.10
Perceived benefits				0.17^†^
Extra physical activities*benefits				0.12*

*P ≤ .05, ^†^ *P* ≤ .01, ^‡^ *P* ≤ .001.

**Table 5:** Hierarchical regression of the physical neighborhood, and home environment on self-reported leisure time sports among boys.

	*R* ^2^	*R*_change_^2^	*F*_change_	
*Block 1*	0.04	0.04	4.38*	*Beta 1*
SES				−0.07
Age				−0.20^†^
*Block 2-Model 2*	0.23	0.19	9.31^‡^	*Beta 1*
Attitude				0.16
Self-efficacy				0.14
Social support				0.02
Perceived benefits				0.19^†^
Perceived barriers				−0.12
*Block 2-Model 3*	0.28	0.23	7.45^‡^	*Beta 2*
Sedentary equipment				−0.07
PA equipment				0.09
Social support				0.03
Self-efficacy				0.25^‡^
Perceived barriers				−0.14
Sedentary equipment*social support				−0.27^‡^
Sedentary equipment*barriers				0.21^†^
PA equipment*self-efficacy				0.12

*P ≤ .05, ^†^ *P* ≤ .01, ^‡^ *P* ≤ .001.

**Table 6:** Hierarchical regression of the physical neighborhood, and home environment on self-reported leisure time sports among girls.

	*R* ^2^	*R*_change_^2^	*F*_change_	
*Block 1*	0.04	0.04	6.53^†^	*Beta 1*
SES				0.09
Age				−0.18^†^
*Block 2-Model 1*	0.05	0.01	3.92*	*Beta 2*
PA equipment				0.11*
*Block 2-Model 2*	0.25	0.21	17.98^‡^	*Beta 1*
Attitude				0.21^‡^
Self-efficacy				0.23^‡^
Social support				0.08
Perceived benefits				0.08
Perceived barriers				−0.07
*Block 2-Model 3*	0.10	0.07	7.78^‡^	*Beta 2*
Perceived barriers				−0.24^‡^
Convenience				0.01
Convenience*barriers				−0.13*

*P ≤ .05, ^†^ *P* ≤ .01, ^‡^ *P* ≤ .001.

**Table 7:** Hierarchical regression of the physical home, neighborhood and school environment on MVPA.

	*R* ^2^	*R*_change_^2^	*F*_change_	
*Block 1*	0.22	0.22	6.14^‡^	*Beta 1*
Gender				−0.49^‡^
SES				0.17
Age				0.06
*Block 2-Model 1*	0.23	0.01	0.57	*Beta 2*
Supervision				0.11
*Block 2-Model 2*	0.40	0.18	3.55^†^	*Beta 2*
Attitude				−0.46^†^
Self-efficacy				0.25
Social support				0.12
Perceived benefits				0.31*
Perceived barriers				0.06
*Block 2-Model 3*	0.32	0.09	2.72*	*Beta 2*
Supervision				−0.09
Self-efficacy				0.03
Supervision*self-efficacy				0.35*

*P ≤ .05, ^†^ *P* ≤ .01, ^‡^ *P* ≤ .001.

## References

[1] Biddle SJH, Gorely T, Stensel DJ (2004). Health-enhancing physical activity and sedentary behaviour in children and adolescents. *Journal of Sports Sciences*.

[2] Sallis JF, Prochaska JJ, Taylor WC (2000). A review of correlates of physical activity of children and adolescents. *Medicine and Science in Sports and Exercise*.

[3] Green LW, Richard L, Potvin L (1996). Ecological foundations of health promotion. *American Journal of Health Promotion*.

[4] Bandura A (1986). *Social Foundations of Thought and Action: A Social Cognitive Theory*.

[5] Sallis JF, Owen N, Glanz K, Rimer BK, Lewis FM (2002). Ecological models of health behavior. *Health Behavior and Health Education*.

[6] Ommundsen Y, Klasson-Heggebø L, Anderssen SA (2006). Psycho-social and environmental correlates of location-sepecific physical activity among 9- and 15-year old
Norwegian boys and girls: the European Youth Heart Study. *International Journal of Behavioral Nutrition and Physical Activity*.

[7] Verstraete SJM, Cardon GM, De Clercq DLR, De Bourdeaudhuij IMM (2006). Increasing children's physical activity levels during recess periods in elementary schools: the effects of providing game equipment. *European Journal of Public Health*.

[8] Ferreira I, van der Horst K, Wendel-Vos W, Kremers S, van Lenthe FJ, Brug J (2006). Environmental correlates of physical activity in youth—a review and update. *Obesity Reviews*.

[9] Sallis JF, Conway TL, Prochaska JJ, McKenzie TL, Marshall SJ, Brown M (2001). The association of school environments with youth physical activity. *American Journal of Public Health*.

[10] Wechsler H, Devereaux RS, Davis M, Collins J (2000). Using the school environment to promote physical activity and healthy eating. *Preventive Medicine*.

[11] McKenzie TL, Marshall SJ, Sallis JF, Conway TL (2000). Student activity levels, lesson context, and teacher behavior during middle school physical education. *American Journal of Preventive Medicine*.

[12] Haug E, Torsheim T, Sallis JF, Samdal O The characteristics of the outdoor school environment associated with physical activity.

[13] Haug E, Torsheim T, Samdal O (2008). Physical environmental characteristics and individual interests as correlates of physical activity in Norwegian secondary schools: the health behaviour in school-aged children study. *International Journal of Behavioral Nutrition and Physical Activity*.

[14] Green K, Smith A, Roberts K (2005). Young people and lifelong participation in sport and physical activity: a sociological perspective on contemporary physical education programmes in England and Wales. *Leisure Studies*.

[15] De Bourdeaudhuij I, Sallis JF, Saelens BE (2003). Environmental correlates of physical activity in a sample of Belgian adults. *American Journal of Health Promotion*.

[16] Humpel N, Owen N, Leslie E (2002). Environmental factors associated with adults' participation in physical activity: a review. *American Journal of Preventive Medicine*.

[17] Cerin E, Vandelanotte C, Leslie E, Merom D (2008). Recreational facilities and leisure-time physical activity: an analysis of moderators and self-efficacy as a mediator. *Health Psychology*.

[18] Hollingshead AB (1957). *Two-Factor Index of Social Position*.

[19] Philippaerts RM, Matton L, Wijndaele K, Balduck A-L, De Bourdeaudhuij I, Lefevre J (2006). Validity of a physical activity computer questionnaire in 12- to 18-year-old boys and girls. *International Journal of Sports Medicine*.

[20] De Bourdeaudhuij I, Sallis J (2002). Relative contribution of psychosocial variables to the explanation of physical activity in three population-based adult samples. *Preventive Medicine*.

[21] De Bourdeaudhuij I, Lefevre J, Deforche B, Wijndaele K, Matton L, Philippaerts R (2005). Physical activity and psychosocial correlates in normal weight and overweight 11 to 19 year olds. *Obesity Research*.

[22] De Bourdeaudhuij I, Philippaerts R, Crombez G (2005). Stages of change for physical activity in a community sample of adolescents. *Health Education Research*.

[23] Puyau MR, Adolph AL, Vohra FA, Butte NF (2002). Validation and calibration of physical activity monitors in children. *Obesity Research*.

[24] Ward DS, Evenson KR, Vaughn A, Rodgers AB, Troiano RP (2005). Accelerometer use in physical activity: best practices and research recommendations. *Medicine and Science in Sports and Exercise*.

[25] Myers RH (1990). *Classical and Modern Regression with Applications*.

[26] Sallis JF (2000). Age-related decline in physical activity: a synthesis of human and animal studies. *Medicine and Science in Sports and Exercise*.

[27] Haerens L, Deforche B, Maes L, Cardon G, Stevens V, De Bourdeaudhuij I (2006). Evaluation of a 2-year physical activity and healthy eating intervention in middle school children. *Health Education Research*.

[28] Haerens L, De Bourdeaudhuij I, Maes L, Cardon G, Deforche B (2007). School-based randomized controlled trial of a physical activity intervention among adolescents. *Journal of Adolescent Health*.

[29] Simon C, Wagner A, DiVita D (2004). Intervention centered on adolescents' physical activity and sedentary behaviour (ICAPS): concept and 6-month results. *International Journal of Obesity*.

[30] Tucker P, Irwin JD, Gilliland J, He M, Larsen K, Hess P (2009). Environmental influences on physical activity levels in youth. *Health and Place*.

[31] Dowda M, McKenzie TL, Cohen DA (2007). Commercial venues as supports for physical activity in adolescent girls. *Preventive Medicine*.

[32] Gordon-Larsen P, Nelson MC, Page P, Popkin BM (2006). Inequality in the built environment underlies key health disparities in physical activity and obesity. *Pediatrics*.

[33] Norman GJ, Nutter SK, Ryan S, Sallis JF, Calfas KJ, Patrick K (2006). Community design and access to recreational facilities as correlates of adolescent physical activity and body-mass index. *Journal of Physical Activity and Health*.

[34] Porter DE, Kirtland KA, Neet JM, Williams JE, Ainsworth BE (2004). Considerations for using a geographic information system to assess environmental supports for physical activity. *Preventing Chronic Disease*.

[35] Olds T, Wake M, Patton G (2009). How do school-day activity patterns differ with age and gender across adolescence?. *Journal of Adolescent Health*.

[36] Cherney ID, London K (2006). Gender-linked differences in the toys, television shows, computer games, and outdoor activities of 5- to 13-year-old children. *Sex Roles*.

[37] Reed JA, Phillips DA (2005). Relationships between physical activity and the proximity of exercise facilities and home exercise equipment used by undergraduate university students. *Journal of American College Health*.

[38] Jago R, Baranowski T, Zakeri I, Harris M (2005). Observed environmental features and the physical activity of adolescent males. *American Journal of Preventive Medicine*.

[39] Verstraete SJM, Cardon GM, De Clercq DLR, De Bourdeaudhuij IMM (2007). A comprehensive physical activity promotion programme at elementary school: the effects on physical activity, physical fitness and psychosocial correlates of physical activity. *Public Health Nutrition*.

[40] Hamilton K, White KM (2008). Extending the theory of planned behavior: the role of self and social influences in predicting adolescent regular moderate-to-vigorous physical activity. *Journal of Sport and Exercise Psychology*.

