# Supplementary material for: The Contribution of Home, Neighbourhood and School Environmental Factors in Explaining Physical Activity among Adolescents
Source: J Environ Public Health. 2009 Sep 14;2009:320372. doi: 10.1155/2009/320372 (PMC2778568; doi:10.1155/2009/320372)
Supplement: Supplementary file 1 — The appendix provides an overview of the selection of questions from the Flemish Physical Activity Questionnaire (Philippaerts et al., 2006) that were used for the present study. [file 320372.f1.pdf]

Appendix 1 (selection of questions) based on the FPAQ (Philippaerts et al., 2006)

By answering the following questions, face a 'usual' week (7 days)

1. **How do you usually go to school and back home?** (indicate the right answer, you can indicate several possibilities)
   - O **cycling**
     How long do you cycle from home to school per day .... minutes
   - O **walking**
     How long do you walk from home to school per day.......... minutes
   - O **by car, train, bus or motorcycle**
     How long does it take to travel from home to school per day .... minutes
   - O **by step or rollerblades**
     How long does it take to travel from home to school per day .... minutes

2. **How much time is weekly provided for PE?** .... minutes per week

3. **In which activities do you participate?** (indicate the right answer, you can indicate several possibilities)
   - O sports or physical activities during lunch break and playtime
   - O sports or physical activities after school hours
   - O sports or physical activities on Wednesday afternoon
   - O sports or physical activities on class- or school tournaments

4. **How much time do you spend on all these activities?** (indicate the right answer)

O now and then	O 2 hours per week
O 1 hour per month	O 3 hours per week
O 2 hours per month	O 4 hours per week
O 3 hours per month	O more than 4 hours per week
O 1 hour per week	

5. **How much time per day do you use walking for transportation in leisure time during a regular weekday?** (the same repeated for cycling)

O 0-10 minutes	O 50-60 minutes
O 10-20 minutes	O 1h-1h10 minutes
O 20-30 minutes	O > 1h10 minutes
O 30-40 minutes	
O 40-50 minutes	

6. **How much time per day do you use walking for transportation in leisure time during a regular weekendday?** (the same repeated for cycling)

O 0-10 minutes	O 50-60 minutes
O 10-20 minutes	O 1h-1h10 minutes
O 20-30 minutes	O > 1h10 minutes
O 30-40 minutes	
O 40-50 minutes	

7. Give the three main sports you practiced most in leisure time.

(note: physical education and school sports do not count for your sports in leisure time)

**My first sport**	(the same for my second and third sport)

O I don't practice a sport in leisure time

O My first sport is: …………………….

<u>How often</u> do you practice this sport? (indicate only 1 option)	<u>How much time (hours)</u> do you spend on this sport?
O now and then	.... hours per year
O one week per year	
O two weeks per year	
O three weeks per year	
O four weeks per year	
O one time per month	.... hours per month
O two times per month	
O three times per month	
O one time per week	.... hours per week
O two times per week	
O three times per week	
O four times per week	
O five times per week	
O six times per week	
O seven times per week	
O more than seven times per week	

Do you participate in organized training?
O yes O no

Do you participate in organized competition?
O yes O no

Thank you very much for your cooperation!!
